# Visualizing Macrophage Polarization through Fluorescent mRNA Profiling

**DOI:** 10.3390/bios14100475

**Published:** 2024-10-02

**Authors:** Miaomiao Xu, Siyuan Wei, Tong Su, Die Ma, Zhixuan Wang, Dan Zhu, Lixing Weng, Xianguang Ding

**Affiliations:** State Key Laboratory of Organic Electronics and Information Displays and Jiangsu Key Laboratory for Biosensors, Institute of Advanced Materials (IAM), Nanjing University of Posts and Telecommunications, 9 Wenyuan Road, Nanjing 210023, China; xumiaomiao@njupt.edu.cn (M.X.); 1022173310@njupt.edu.cn (S.W.); 2023060726@njupt.edu.cn (T.S.); 1223066421@njupt.edu.cn (D.M.); 2023060731@njupt.edu.cn (Z.W.);

**Keywords:** macrophages, mRNA, cellular imaging

## Abstract

Macrophages, known for their phenotypic plasticity, play a critical role in maintaining homeostasis and inflammation-related pathogenesis. Although identifying diverse macrophage phenotypes holds promise for enhancing diagnoses and treatments of diseases mediated by macrophages, existing methodologies for differentiating macrophages often lack precision. They are limited by the cumbersome procedures that require large-scale equipment, such as flow cytometry and transcriptomic analysis. In this context, we have engineered fluorescent polyadenine (polyA)-mediated sticky flares that enable practical visualization of macrophages. This technology facilitates the highly sensitive detection of macrophage phenotypes through the specific recognition of intracellular mRNAs, permitting in situ imaging. Our approach demonstrates the potential for determining macrophage polarization status at the single-cell level within dynamic immune microenvironments, thereby providing crucial diagnostic and prognostic information that could guide the development of tailored treatments for macrophage-related diseases in personalized medicine.

## 1. Introduction

Macrophages exhibit the ability to adapt their physiology in response to environmental cues, manifesting as the activation of distinct phenotypes: pro-inflammatory (M1) and anti-inflammatory (M2). These differentially activated macrophages are essential for both homeostasis and the pathogenesis of inflammation-related conditions, including autoimmune disorders, cardiovascular diseases, and cancer [1,2,3]. M1 macrophages, characterized by high expression of CD86, initiate an inflammatory response by upregulating the expression of pro-inflammatory genes, such as nuclear transcription factor-κB (NF-κB), leading to the release of inflammatory cytokines like IL-1β and TNF-α. These cytokines actively contribute to the promotion and rupture of unstable atherosclerotic plaques, pathogen defense, and tumor suppression, primarily through the activation of both innate and adaptive immune responses. M2 macrophages, characterized by high expression of mannose receptor (also known as CD206), IL-1 receptor (IL-1R), and C-C motif chemokine ligand 17 (CCL17), secrete profibrotic factors and exhibit elevated arginase-1 (ARG-1) activity. These unique traits enable M2 macrophages to participate in a range of physiological and pathological processes, including pathogen and parasite clearance, anti-inflammatory responses, wound healing, tissue remodeling, and immune regulation. However, they are also implicated in promoting an immunosuppressive tumor microenvironment that supports tumor progression [4,5]. Consequently, characterizing macrophage phenotypes and processes offers significant promise for advancing diagnostic techniques across multiple diseases and enhancing the targeted evaluation of macrophage immune responses in therapeutic interventions [6].

Macrophages exhibit a high degree of heterogeneity, driven by complex molecular mechanisms that facilitate their adaptation. Throughout various stages of the same disease and influenced by other conditions in the host, macrophages continuously encounter microenvironments filled with diverse stimuli, leading to unpredictable activation along the M1-M2 polarization spectrum [7,8,9]. Furthermore, the development of molecular imaging technologies specifically targeting macrophages and their markers can enable early detection and diagnosis of diseases such as atherosclerosis and cancer. Moreover, this will facilitate the identification of lesion sites and provide possibilities for subsequent targeted therapies. Challenges in clinical applications include the variability of individual immune responses, the difficulty in visually confirming positive patient responses to immunotherapies, and the absence of fast, accurate, and direct methods to monitor macrophage activity through markers of various polarization types. These obstacles have significantly limited research into macrophage-related diseases across multiple contexts [10,11].

The ability to monitor and visualize mRNA content and distribution within live cells provides critical insights into cellular heterogeneity, essential for advancing clinical research [12,13,14,15]. Traditional methods for mRNA analysis require cell disruption and RNA extraction, which are complex and costly, compromise cell integrity and biological relevance of the targeted RNA and raise safety concerns. In response, we have developed a fluorescent nanoprobe utilizing gold (Au) nanoparticles. This combines the specific molecular recognition and programmability of nucleic acids with the optical properties of fluorescent gold nanoparticles (AuNPs) to aid in diagnosing macrophage polarization phenotypes [16,17,18]. The procedure involves the utilization of a DNA sequence specifically designed to recognize mRNA associated with polarized macrophages as a binding intermediary. This sequence acts as a scaffold to anchor a fluorescent reporter strand onto the AuNPs’ surface. The resultant fluorescent probe, which is specific to the mRNA markers of polarized macrophages, is synthesized by exploiting the fluorescence amplification characteristic of AuNPs. This synthesis achieves a detection method that is not only highly selective and sensitive but also maintains the structural and functional integrity of the targeted mRNA.

## 2. Material and Methods

### 2.1. Materials and Reagents

The CCK-8 cell proliferation assay kit and DMEM (high glucose) medium (including double antibiotic) were purchased fromJiangsu KeyGen Biotechnology Co. (Nanjing, China), Ltd. RPMI-1640 (including double antibiotic) was purchased from Jiangsu KeyGen Biotechnology Co. (Nanjing, China), Ltd. Lipopolysaccharide (LPS) Phorbol was purchased from Biyuntian Biotechnology (Shanghai, China). 12-myristate 13–acetate (PMA) was purchased from Sigma (St. Louis, MO, USA). Recombinant Murine IL-4, Recombinant Human IL–4, Recombinant Human IL-13, and Recombinant Human IFN-γ were purchased from Shanghai Dakewei Biotechnology Co. (Shanghai, China), Ltd. Sodium hydrogen phosphate dodecahydrate (Na_2_HPO_4−_12H_2_O), sodium dihydrogen phosphate dihydrate (NaH_2_PO_4−_2H_2_O), sodium chloride (NaCl), and Tris were purchased from Sinopharm Holding Chemical Reagent Company Limited (Shanghai, China). Deoxyribonuclease I (DNase I) and all base sequences were synthesized, and HPLC was purified by Shanghai Sangon Biotech. (Shanghai, China). See Table 1.

### 2.2. Nanoprobe Assembly and Feasibility Detection

The mRNA in situ detection nanoprobes for typing the polarization state of macrophages were prepared using the “salt aging” method [19,20,21]. First, the DNA recognition sequence with polyadenine (polyA) tail modification corresponding to the polarized RAW264.7 macrophage mRNA was mixed with 10 nm AuNPs at a molar ratio of 150:1 and incubated in a thermostatic oscillator at 25 °C for 12 h. Subsequently, 1 M PBS (100 mM PB, 1 M NaCl, pH = 7.4) was added in 10 batches (15 min apart) to reach a final PBS concentration of 0.1 M (10 mM PB, 1 M NaCl, pH = 7.4) so that the final concentration of PBS in the mixed solution was 0.1 M (10 mM PB, 0.1 M NaCl, pH = 7.4). After incubation for 12 h in a constant temperature oscillator at 25 °C, the supernatant was removed by centrifugation at 12,000 rpm, 20 min, and 15 °C centrifugation parameters. Then, 0.1 M PBS solution was added, and the centrifugation was repeated and washed thrice to ensure that AuNPs–DNA composite probes were obtained without free DNA recognition sequences. The AuNPs–DNA hybrid probes were dispersed in Tris buffer (20 mM Tris, 300 mM NaCl, pH = 8.0). Subsequently, the Cy5 fluorescently labeled CD80/CD206 fluorescent reporter strands (final concentration of 500 nM each) were mixed with the obtained DNA-AuNPs (final concentration of 5 nM). The experiment was conducted in a temperature-controlled oscillator, shielded from light, for a period of 3 h at 37 °C and 300 rpm. The supernatant was discarded according to the same centrifugation conditions and washed and centrifuged three times using Tris buffer to remove unhybridized free fluorescent reporter chains and obtain the pure fluorescent nanoprobe. Concentrations were calculated according to the Lambert–Beer law. The final probe was dispersed in Tris buffer and stored at 4 °C in an environment protected from light for short-term use. The M1-type and M2-type RAW264.7 macrophage polarization markers CD80 and CD206 sequences were used as target sequences for in vitro feasibility testing. The polyA-mediated nanoprobes were diluted in Tris buffer (20 mM Tris, 200 mM NaCl, pH = 8.0), and 200 nM CD80-T and CD206-T target sequences were added, respectively. The reaction solution was collected using a micro quartz cuvette after incubation for 2 h at 37 °C under light protection and tested by fluorescence spectrometer at 680–800 nm.

### 2.3. Specificity Detection

The polyA-mediated polarized macrophage nanoprobes with a final concentration of 8 nM were dispersed in Tris buffer and mixed with different sequences. These nanoprobes with mixtures were incubated in a constant temperature oscillator at 37 °C for 2 h, protected from light, and 70 μL of the reaction mixture solution was taken into a micro quartz cuvette. The polyA-mediated nanoprobes, with a concentration of 8 nM, were dispersed in Tris buffer, combined with various sequences, and incubated in a constant temperature oscillator at 37 °C, shielded from light, for 2 h. A total of 70 μL of the reaction mixture solution was then transferred to a micro quartz cuvette. The fluorescence intensities of the groups were excited and measured. The results were subjected to a nonlinear regression curve fitting.

### 2.4. Nuclease Stability and Biocompatibility

The polarized macrophage nanoprobes with a concentration of 8 nM were dispersed in Tris buffer (20 mM Tris, 200 mM NaCl, pH = 8.0), and the mixture was divided into two groups. In each group, the DNase I enzyme was added at a concentration of 2 U/L. As a control, the other group received an equal volume of Tris solution, and the fluorescence signals of the probes were monitored every 2 h from 0 to 12 h. The fluorescence signals were measured in 2 h intervals from 0 to 12 h, and the changes in fluorescence were then graphed. When the nanoprobes were incubated with the corresponding 200 nM CD80-T and CD206-T target sequences, 2 U/L of DNase I enzyme and an equal volume of Tris buffer were added, and the mixture was incubated for 2 h at 37 °C in a constant temperature oscillator, under the protection of light. An adequate amount of the mixed solution was obtained and placed in a micro quartz cuvette. The fluorescence intensities of the two sets of conditions were measured and stored.

To evaluate the biocompatibility of the probe, cytotoxicity was performed using a CCK-8 assay. RAW264.7 macrophages were spread in 96-well cell culture plates at a concentration of 1 × 10^4^ cells/well overnight, with 6 replicate wells in each group. A blank group was set up containing only culture medium and no cells; the original cell culture medium was discarded, and the cells were washed gently with PBS three times. For each cell well, 1 and 5 nM concentrations of gold nanoparticles and nanoprobes were added in equal volumes, and the cells were incubated for 12, 24, and 48 h. After incubation for 12, 24, and 48 h, the original culture medium was removed, and the cells were washed gently with PBS three times. Subsequently, 100 μL of fresh culture medium and 10 μL of CCK-8 reagent were added to the wells of each cell group. Finally, the absorbance at 450 nm was read by an enzyme marker.

### 2.5. Cellular Imaging

Stimulating RAW264.7 cells M0-type into M1-type with 100 ng/mL LPS for 24 h. Stimulating RAW264.7 cells M0-type into M2-type with 10 ng/mL Recombinant Murine IL-4 for 24 h.

Treating THP-1 cells with 5 ng/mL PMA for 48 h to induce THP-1 to macrophages. Stimulating THP-1 cells into M1-type with 100 ng/mL LPS and 20 ng/mL Recombinant Human IFN-γ for 48 h. Stimulating THP-1 cells into M2-type with 20 ng/mL Recombinant Human IL-4 and 20 ng/mL Recombinant Human IL-13 for 48 h.

Cells of each group were inoculated in 15 mm confocal cell culture dishes. After 24 h, when the cells were adhered to the wall and reached the logarithmic growth phase, the medium was refreshed, and various fluorescent nanoprobes were introduced at a final concentration of 1 nM. The cells were incubated in darkness for 3 h before the supernatant was removed. Subsequently, the cells at the bottom of the dishes were covered with a PBS solution and gently shaken three times to wash away any un-endocytosed probes. Fluorescence excitation of Cy5 was carried out using fluorescence confocal microscopy to observe the fluorescence imaging results. The cells utilized for these experiments were sourced from the ATCC cell bank.

## 3. Results and Discussion

**Scheme 1:**
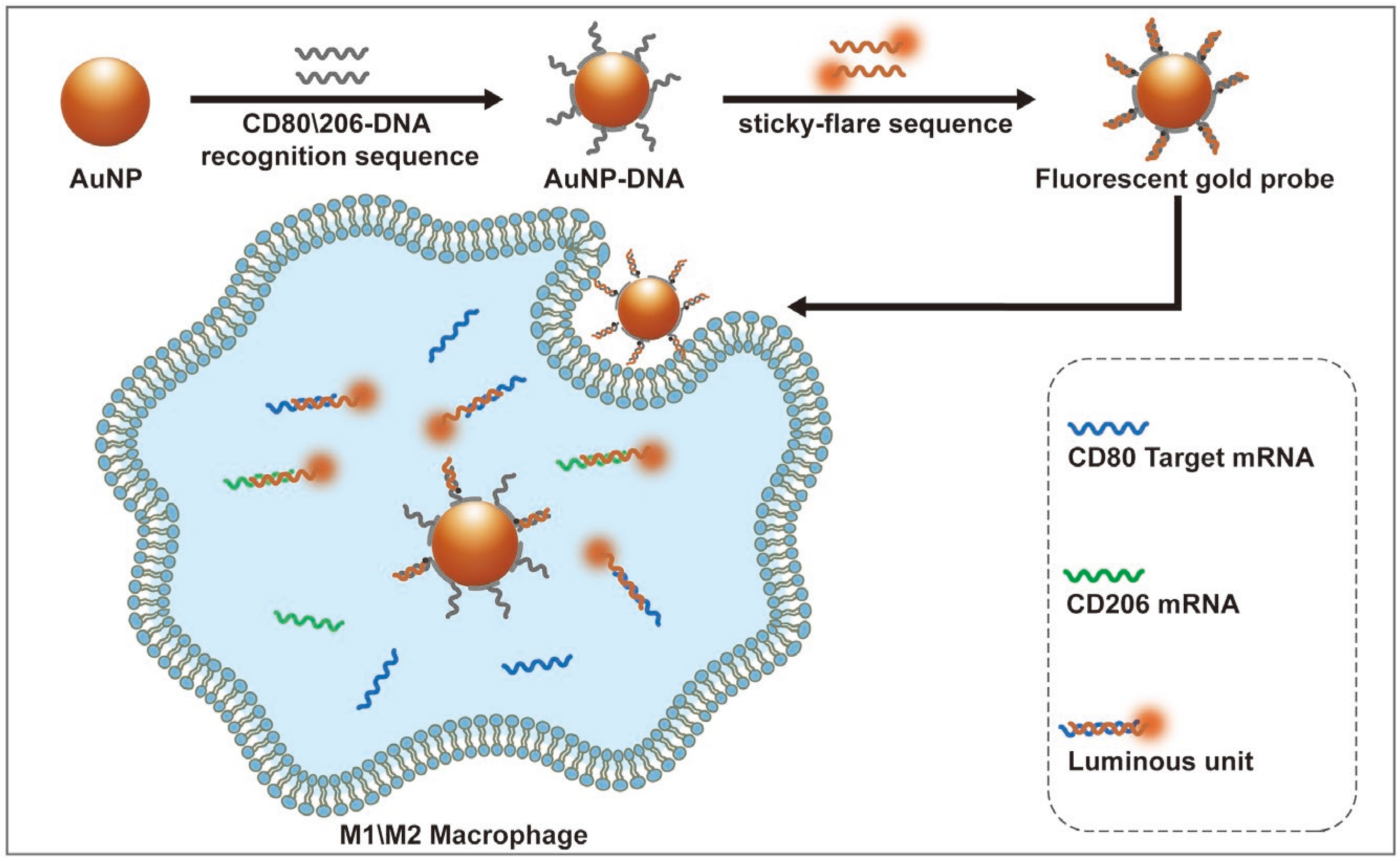
Schematic illustration of the fluorescent gold nanoprobes used to detect polarization-associated mRNAs in macrophages.

In this study, we leveraged the distinct mRNA profiles during the maturation and activation of mouse RAW264.7 macrophages to create an AuNPs–DNA nanocomposite probe. This was achieved through the self-assembly of polyA-modified DNA strands on the surface of AuNPs (Scheme 1). To identify polarized macrophages, a fluorescent moiety was hybridized with a Cy5-labeled adhesive reporter strand that was complementary to CD80/CD206 mRNA, forming a fluorescent probe. Through the competitive base complementary pairing of intracellular specific mRNA with the fluorescent reporter chain, the fluorescent chain detached from the gold spheres was able to restore fluorescence, leading to high sensitivity, specificity, and stability in fluorescence detection of in vitro polarized macrophage target sequences, as well as in situ imaging of M1-polarized macrophages under simulated inflammatory conditions. This technique has the potential to detect macrophage polarization status at the single-cell level in dynamic immune microenvironments, including tumors, wounds, and cardiovascular diseases. It provides critical diagnostic and prognostic insights, aiding in the quantification of therapeutic efficacy and the development of enhanced treatments, thus advancing personalized clinical care for macrophage-associated diseases.

### 3.1. Design and Establishment of the Fluorescent mRNA Probe

**Figure 1:**
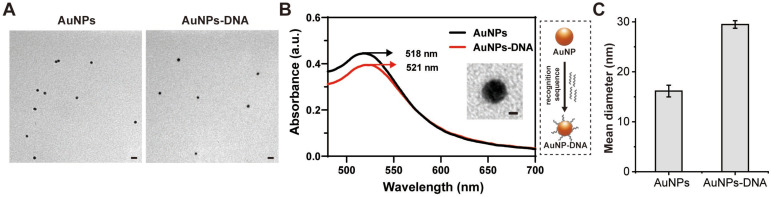
(**A**) TEM images of AuNPs and AuNPs–DNA. Scale bar: 20 nm. (**B**) UV absorbance of AuNPs–DNA and magnified TEM image of individual AuNPs–DNA. Scale bar: 5 nm. (**C**) DLS analysis of AuNPs and AuNPs–DNA.

It has been reported that smaller-sized spherical AuNPs show higher macrophage uptake efficiency compared to larger nanoparticles and that bare gold nanomaterials free of surface modifications of immunostimulatory molecules are biocompatible, non-immunogenic, and essentially polarity-activated for macrophages. Additionally, AuNPs are resistant to degradation and can enter cells without the need for transfection agents [22,23,24,25]. Therefore, we chose to use 10 nm-sized bare AuNPs as the carrier material for the nanoprobes and achieved the preparation of fluorescent nanoprobes in two steps. Firstly, DNA recognition sequences with polyA tails were assembled onto AuNPs. PolyA serves as an effective anchoring block that preferentially adheres to the surface of AuNPs, while an additional recognition block promotes the upright orientation of DNA hybridization. DNA, known for its inherent recognition capabilities and its structural and functional diversity, can be combined with synthetic polymers to construct gold nanoparticles into multifunctional nanostructures [26]. Figure 1A shows the TEM images of AuNPs and AuNPs–DNA. The UV-vis spectra of the AuNPs–DNA composite probes obtained by centrifugal washing (Figure 1B) show that the absorption peak of the unmodified bare 10 nm spherical AuNPs at 518 nm, whereas that of the assembled AuNPs–DNA with the addition of the DNA recognition sequence appeared at 521 nm, with the position of the absorption peak being slightly red-shifted. The red shift in the spectrum confirms the successful functionalization of AuNPs with DNA, as the position of the plasmon band of metal nanoparticles is closely related to their size and surrounding environment [27]. Additionally, DLS analysis revealed an increase in particle size following assembly, confirming the successful attachment of DNA onto the AuNPs (Figure 1C).

**Figure 2:**
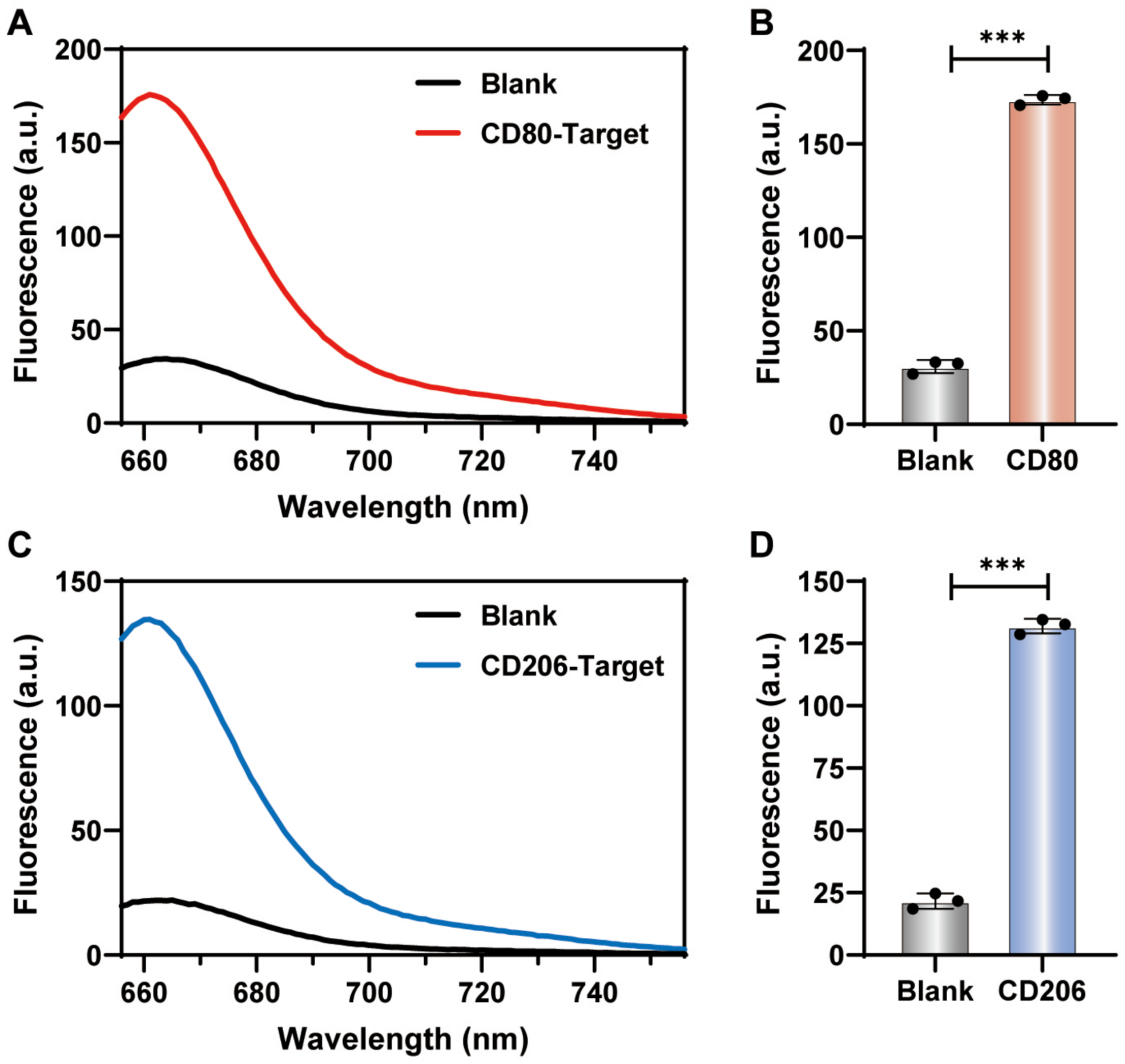
Detection feasibility of fluorescent mRNA probe. (**A**,**B**) Fluorescence emission spectra of the mRNA probe and corresponding quantitative analyses in the presence or absence of the target M1 macrophage. (**C**,**D**) Fluorescence emission spectra of fluorescent probe and complementary quantitative studies in the presence or absence of the M2 macrophage CD206 target. Data are expressed as the mean ± SEM (n = 3) (*** *p* < 0.001).

After the formation of the AuNPs–DNA (CD80/CD206) composite probe, we hybridized and connected it with a Cy5 fluorescent motif labeling, assembling a fluorescent nanoprobe for polarized macrophages. Based on the mRNA base information of CD80 and CD206 from polarized macrophages, one of the characteristic sequences was selected to design and synthesize a homologous DNA target sequence, which was used as the target sequence for in vitro feasibility verification (CD80/206-Target), and the feasibility of luminescence was verified by fluorescence spectrometry after the fluorescent probe was incubated with the target sequence for 2 h under light (Figure 2). In the absence of a target sequence, the fluorescence of the probe was quenched due to the FRET effect between Cy5 and AuNPs [28,29]. Simultaneously, the CD80 fluorescent nanoprobe of the M1 macrophage and the CD206 fluorescent nanoprobe of the M2 macrophage could bind to and fluoresce with their corresponding DNA target sequences. The fluorescence signal of the CD80 target sequence group was 5.69 times that of the background fluorescence signal. The fluorescence signal of the CD206 target sequence group could reach 6.22 times the background fluorescence signal, indicating that the prepared probes were feasible and had good target responsiveness and sequence recognition.

### 3.2. Sensitivity of Fluorescent mRNA Probes In Vitro

**Figure 3:**
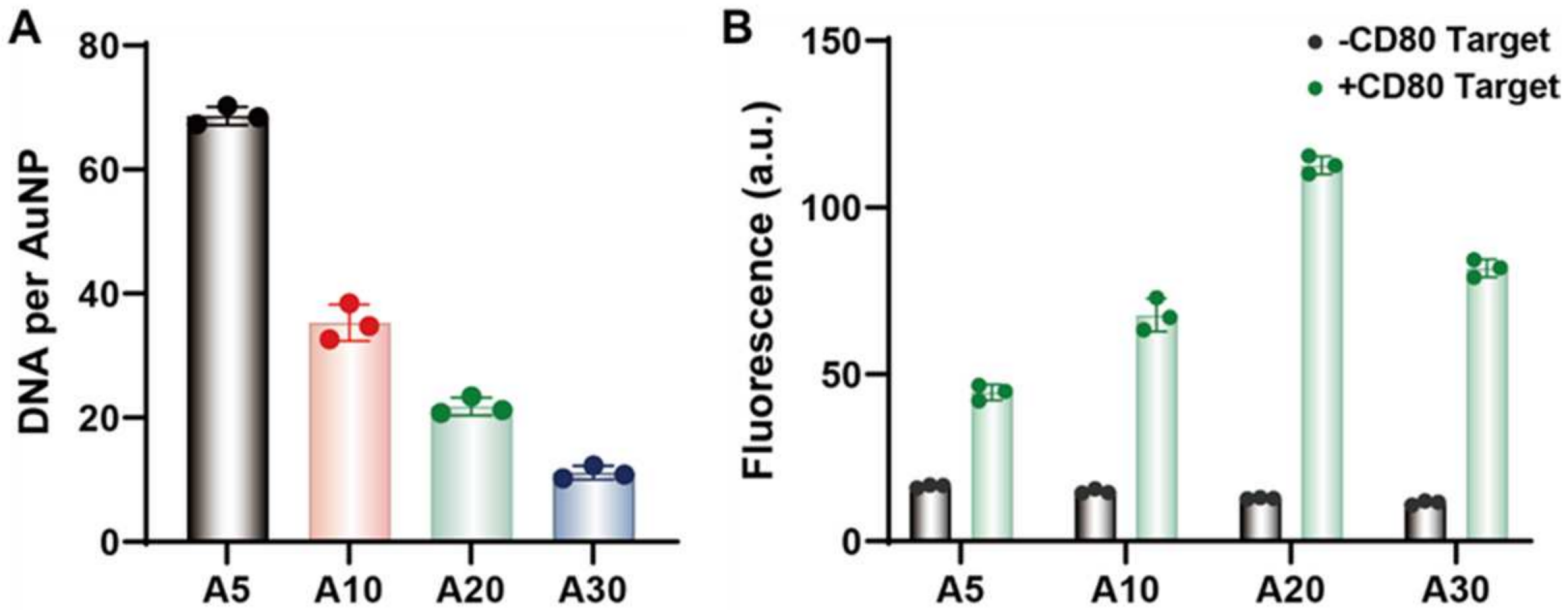
(**A**) The loading amount of fluorescent DNA tracks on gold nanoprobes with polyA tails from A5 to A30. (**B**) The fluorescence of M1-CD80 nanoprobe in the absence and presence of 200 nM CD80 (CD80-T) target sequence at 37 °C for 3 h incubation.

The assembly density of DNA probes on the surface of AuNPs significantly impacts the efficiency of hybridization and biomolecule recognition, thus necessitating an investigation into the effect of varying polyA lengths (ranging from A5 to A30) on these parameters (Figure 3A). The loading density of gold nanoprobes was determined using a fluorescence-based method [30], revealing a gradual decrease with increasing polyA length. Among polyA5, A10, A20 and A30, the polyA20-mediated nanoprobes have the best signal-to-noise ratio for mRNA detection, indicating that maintaining DNA assembly at an appropriate density enhances subsequent hybridization with fluorescent probes (Figure 3B). Therefore, polyA20 was selected as the optimal length for the chimeric macrophage nanoprobe.

**Figure 4:**
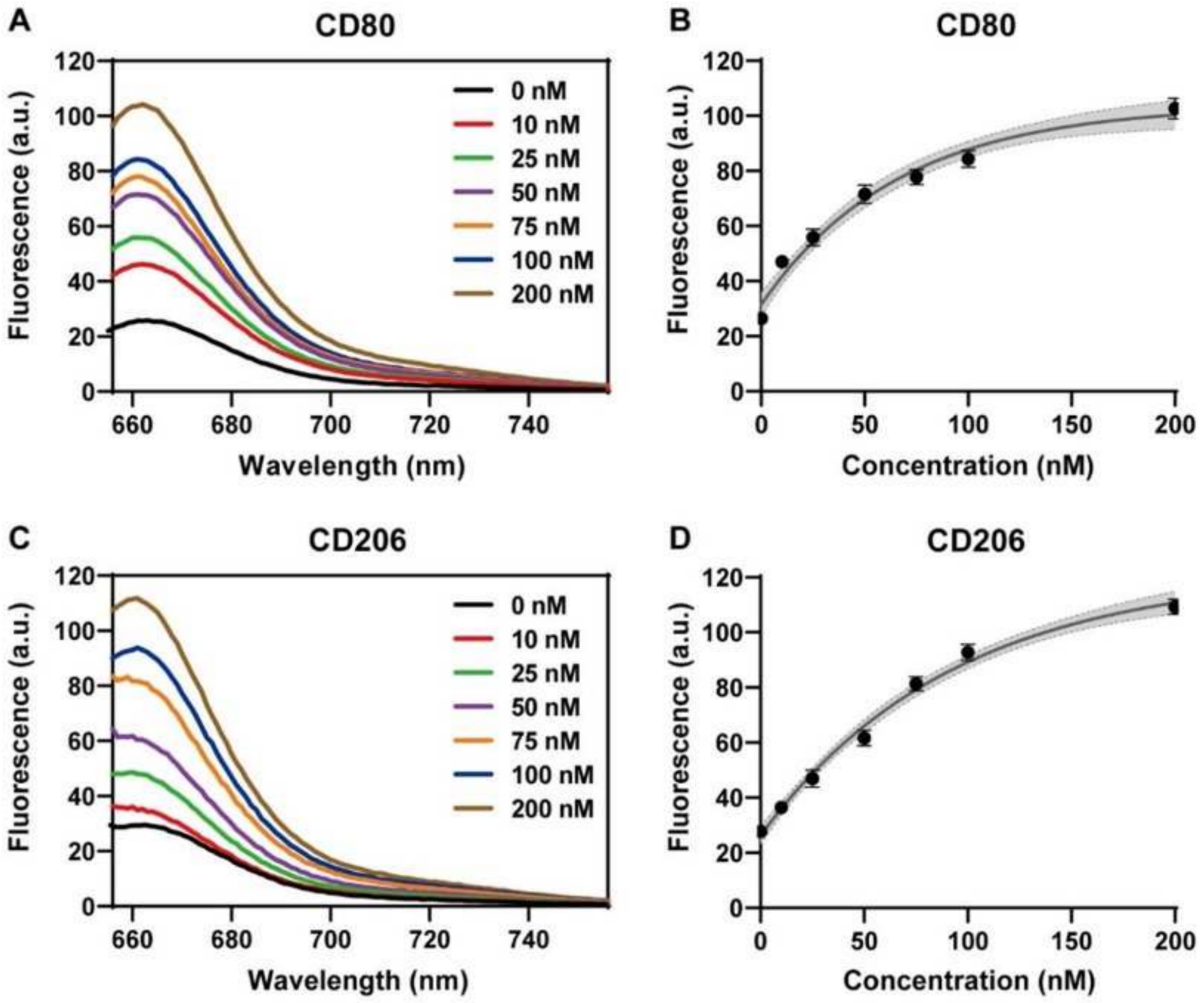
Sensitivity to polyA20-mediated concentration detection of Cy5-labeled fluorescent nanoprobes. (**A**,**B**) Fluorescence spectra and corresponding fluorescence intensity-concentration curves under 646 nm excitation after adding the 0–200 nM M1 macrophage CD80-T target sequence. (**C**,**D**) Fluorescence spectra and corresponding fluorescence intensity-concentration curves under 646 nm excitation after adding 0–200 nM M2 macrophage CD206-T target sequence. All experiments were repeated more than three times, in which the fluorescence intensity-concentration curves were analyzed by nonlinear regression at a 95% confidence level.

We evaluated the detection sensitivity of the probe for DNA target sequences in vitro. A fluorescent gold nanoprobe at a concentration of 4 nM was incubated with CD80/CD206 target sequences at a range of 0–200 nM for 2 h in the absence of light. The fluorescence intensity of the resulting solution was measured with 646 nm excitation (Figure 4). After the addition of the CD80-T and CD206-T target sequences, the fluorescent probe produced fluorescence signals. The fluorescence peak at 664 nm increased in intensity as the concentration of the target sequences increased, indicating that the probe’s fluorescence reporter chain could identify and detach the corresponding target sequences from the gold nanoprobe, thereby restoring the fluorescence. The least-squares method was applied to nonlinearly regress the fluorescence intensity-concentration curves. The results showed a strong association between the fluorescent signals generated by the polyA20-mediated nanoprobe and the concentrations of the target sequences in the CD80-T and CD206-T targets. Figure 4 displays a significant change in the signal based on the target concentrations, illustrating that the fluorescence intensity is proportional to the target concentrations.

**Figure 5:**
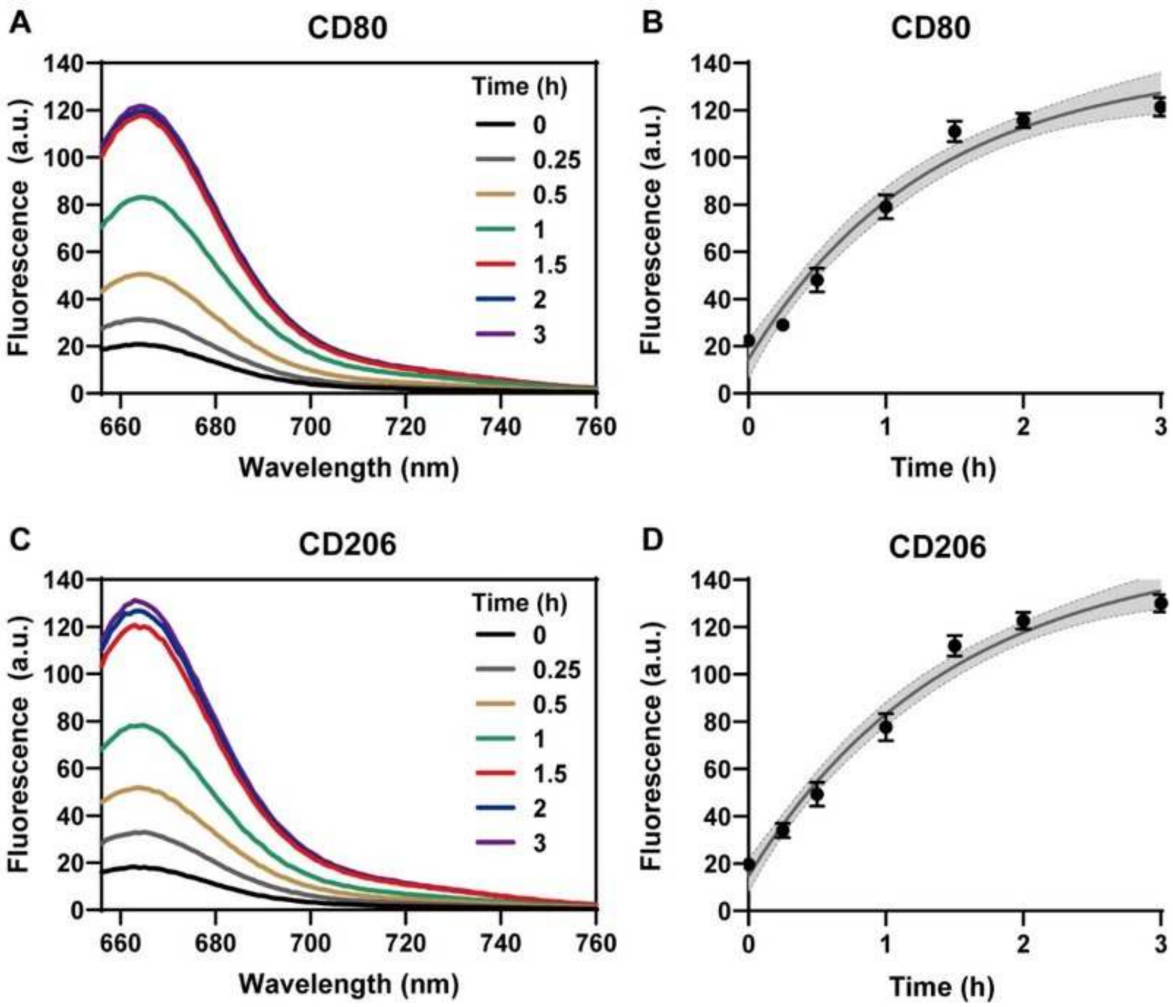
Sensitivity of polyA20-mediated temporal detection of Cy5 fluorescent gold nanoprobes. (**A**,**B**) Fluorescence spectra and corresponding fluorescence intensity-time curves under 646 nm excitation after addition of 200 nM M1 macrophage CD80-T target sequences incubated for 0–3 h. (**C**,**D**) Fluorescence spectra and corresponding fluorescence intensity-time curves under 646 nm excitation after the addition of 200 nM M2 macrophage CD206-T target sequences incubated for 0–3 h. All experiments were repeated more than three times, in which the fluorescence intensity-time curves were analyzed by nonlinear regression at a 95% confidence level. (**C**,**D**) Fluorescence spectra and corresponding fluorescence intensity-time curves after addition of 200 nM M2 macrophage CD206-T target sequence incubated for 0–3 h under 646 nm excitation; all experiments were repeated more than three times, in which the fluorescence intensity-time curves were analyzed by nonlinear regression with a confidence level of 95%.

To evaluate the time response sensitivity of the fluorescent nanoprobe, a target sequence was reacted with the probe at a concentration of 4 nM for different time durations ranging from 0 min to 3 h (0 min, 15 min, 30 min, 1 h, 1.5 h, 2 h, and 3 h) at a concentration of 200 nM (Figure 5). Under 646 nm excitation, the fluorescence signal of the CD80/CD206 gold nanoprobe was enhanced with an increased incubation time with the CD80-T/CD206-T target sequence and exhibited a fluorescence response as early as 15 min of incubation. When the incubation length exceeded 1.5 h, the fluorescence intensity reached a plateau, indicating that the probe-target concentration had stabilized at the probe-target ratio of 1.5 h. At this rate, either the probe was exhausted or the target detection had reached saturation, causing no more growth of fluorescence intensity after 1.5 h. The nonlinear regression curves of fluorescence intensity and time revealed that the M1-CD80 and M2-CD206 fluorescent gold nanoprobes had a time-dependent detection of target sequences, exhibiting an impressive time-responsive sensitivity that could be detected after just 15 min of incubation.

### 3.3. Specificity Analysis of Fluorescent mRNA Probes for Macrophage Detection

**Figure 6:**
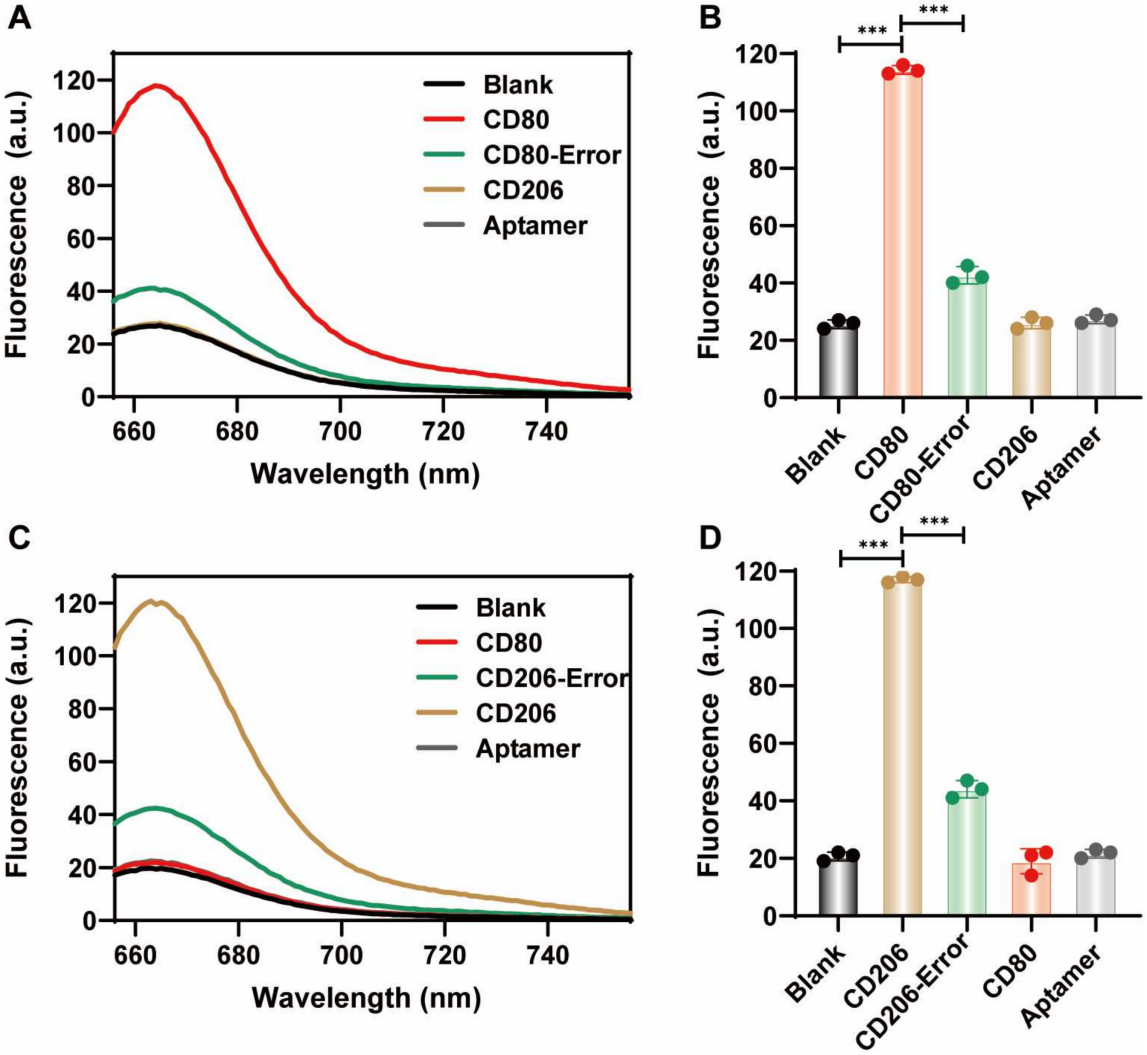
Detection specificity of Cy5 fluorescent fluorescent nanoprobes. (**A**,**B**) M1-CD80 nanoprobe with an equal volume of Tris buffer (Blank no target), 200 nM CD80 (CD80-T) target sequence, 200 nM CD80-Error target single base mismatch sequence, and 200 nM CD206 (CD206-T) sequence. Fluorescence response spectra of Aptamer sequences under 646 nm excitation after incubation for 3 h and the corresponding quantitative results. (**C**,**D**) M2-CD206 nanoprobe with an equal volume of Tris buffer (Blank without target), 200 nM CD206 (CD206-T) target sequence, 200 nM CD206-Error target single base mismatch sequence. The fluorescence response spectra of 200 nM CD80 (CD80-T) and Aptamer sequences under 646 nm excitation after incubation for 3 h. All experiments were repeated three times. Data are expressed as the mean ± SEM (n = 3) (*** *p* < 0.001).

The fluorescent nanoprobes’ specificity depends on the base sequences of the detection substrates, as they identify the target sequences based on the Watson-Crick base complementary pairing principle. To further investigate the base interference resistance and detection specificity of the fluorescent gold nanoprobes, we tested the probes’ fluorescence output signals when exposed to different base sequences. We selected the CD80-T/CD206-T target strand, which is entirely complementary to the Cy5 fluorescent nanoprobe for M1-CD80/M2-CD206 macrophages, the CD80-Error/CD206-Error DNA strand, which is a single-base mismatch concerning the target sequence, and a piece of an unrelated aptamer sequence as the different substrate sequences for the assay. We used the CD80-T and CD206-T sequences cross-paired with CD206 and CD80 for incubation to mimic the complex polarized environment of macrophage populations containing both M1 and M2 phenotype cells. The fluorescence spectra were tested and quantified after 3-h incubation in the dark. As illustrated in Figure 6, the nanoprobes emitted strong fluorescence only in response to their specific target sequences. When a single base mismatch occurred, the fluorescence intensity was slightly higher than the background fluorescence observed in the negative control.

In contrast, no significant fluorescence signals were observed for cross-pairing the CD80-T and CD206-T sequences or for irrelevant random adaptor sequences that did not show substantial fluorescence response. Their fluorescence response curves overlapped with the blank control group’s background fluorescence signal curves. These experimental results indicate that the macrophage Cy5 fluorescent nanoprobe has high selectivity and specificity for the corresponding target sequences.

### 3.4. Nuclease Stability and Cytotoxicity of Nanoprobes

**Figure 7:**
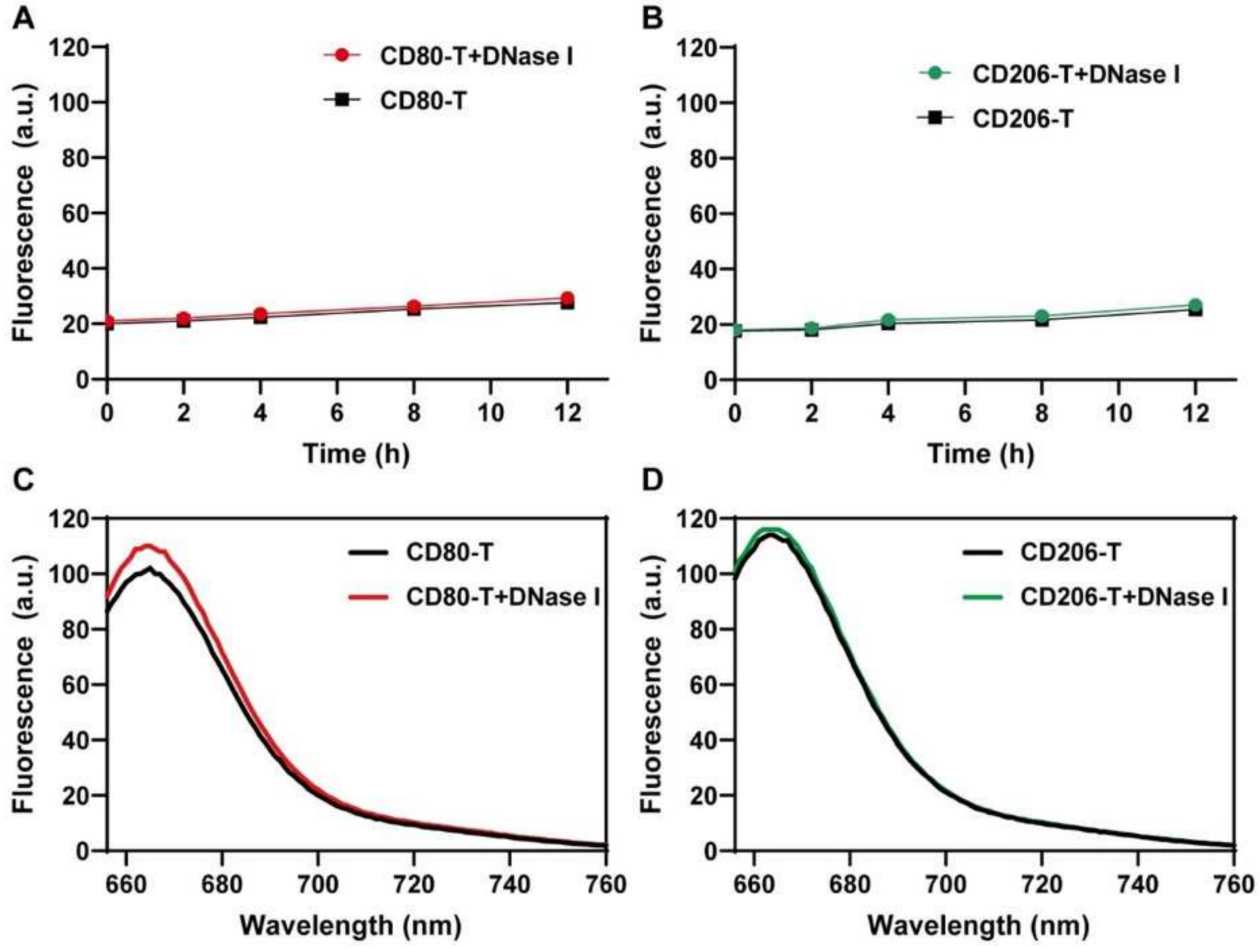
Nuclease stability of Cy5 fluorescent nanoprobes. (**A**,**B**) Background fluorescence signal change curves of M1-CD80 or M2-CD206 nanoprobes with and without DNase I enzyme over 0–12 h. (**C**,**D**) Fluorescence response of M1-CD80 or M2-CD206 nanoprobes with and without DNase I enzyme for target sequences at 200 nM concentration.

To assess the applicability of polyA20-mediated fluorescent gold nanoprobes for macrophage polarization detection, we evaluated the stability and cytotoxicity of the fluorescent probe. Since the primary functional components of the study are DNA recognition and reporter strands, there is a potential risk of enzymatic degradation within living cells. Therefore, we first simulated the enzymatic digestion environment under physiological conditions in vitro and selected a common endonuclease (DNase I), a nucleic acid endonuclease capable of degrading both single-stranded and double-stranded DNA to produce either single deoxynucleotides or single- or double-stranded oligodeoxynucleotides, to test the degree of the fluorescent gold nanoprobe’s tolerance to the nuclease [31], and to assess the probe’s nuclease based on 646 nm-excited Cy5 fluorescence spectroscopy. We tested the background fluorescence signals of the probes when they were left to stand for 0–12 h, protected from light under conditions with or without DNase I enzyme in the system (Figure 7A,B). The experimental results indicated that the background fluorescence of the fluorescent gold nanoprobes treated with DNase I for 12 h did not vary significantly from the non-DNase I-treated probes. Furthermore, the alteration in the background fluorescence signals of the two systems within 12 h was indistinguishable. Non-DNase I-treated nanoprobes were used as a control. When the target sequences of M1-CD80 or M2-CD206 nanoprobes were added to the endonuclease-treated and non-endonuclease-treated probe systems, respectively, a significant fluorescence enhancement was observed under the excitation of 646 nm, demonstrating the stability of the probes’ detection effect under endonuclease conditions (Figure 7C,D). This result indicates that the probe’s detection capability is preserved even in the presence of nucleic acid endonucleases. The introduction of DNase I did not compromise the performance of the macrophage fluorescent gold nanoprobe. The negatively charged AuNPs, which serve as the carrier for the nanoprobes, along with the high local salt concentration near the nanoparticles’ surface, may protect the nucleic acid strands assembled and hybridized on them from degradation by DNase I. Additionally, it was confirmed that the probe’s fluorescence signals are derived from the fluorescence recovery upon desorption of the sticky fluorescent reporter strand from the gold particles after hybridization with the target strand through base complementary pairing rather than from degradation by the nuclease.

**Figure 8:**
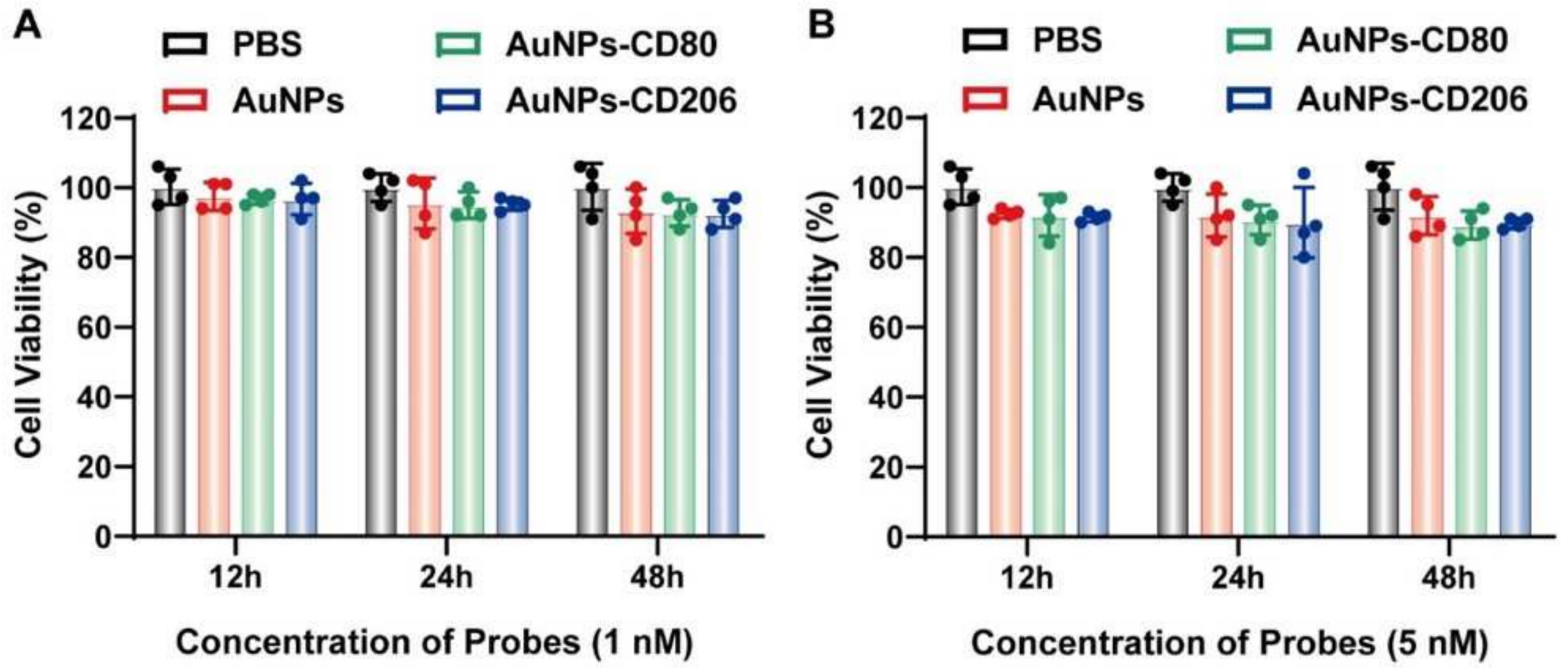
Cytotoxicity of Cy5-labeled nanoprobes. (**A**,**B**) Cellular activity of equal volumes of PBS, naked AuNPs at final concentrations of 1 or 5 nM, and M1-CD80 or M2-CD206 nanoprobes at final concentrations of 1 or 5 nM after co-incubation for 12, 24, and 48 h with mouse RAW264.7 macrophages.

Prior to utilizing the fluorescent nanoprobe for macrophage detection, we evaluated its impact on macrophage activity using the CCK-8 reagent. A PBS solution served as a blank control. Unmodified naked AuNPs at concentrations of 1 and 5 nM, as well as M1-CD80 and M2-CD206 fluorescent gold nanoprobes (labeled as AuNPs-CD80 and AuNPs-CD206, respectively), were incubated with logarithmically growing, uncontaminated, healthy RAW264.7 macrophages for periods of 12, 24, and 48 h. We then measured the cell activity for each group. By normalizing the experimental data to the cell viability of the PBS blank control group, as presented in Figure 8, it was found that neither the unmodified gold particles nor the modified fluorescent gold nanoprobes exhibited toxic effects on the living cells within 48 h. The macrophage activity in each group remained stable at 90% and above, indicating that the fluorescent gold nanoprobes developed for the macrophage polarization typing assay were biocompatible. The results from nuclease stability experiments and cytotoxicity tests confirm that the probe can detect living cells without inducing toxicity or reducing their stability.

### 3.5. Intracellular Imaging with Fluorescent Probes

**Figure 9:**
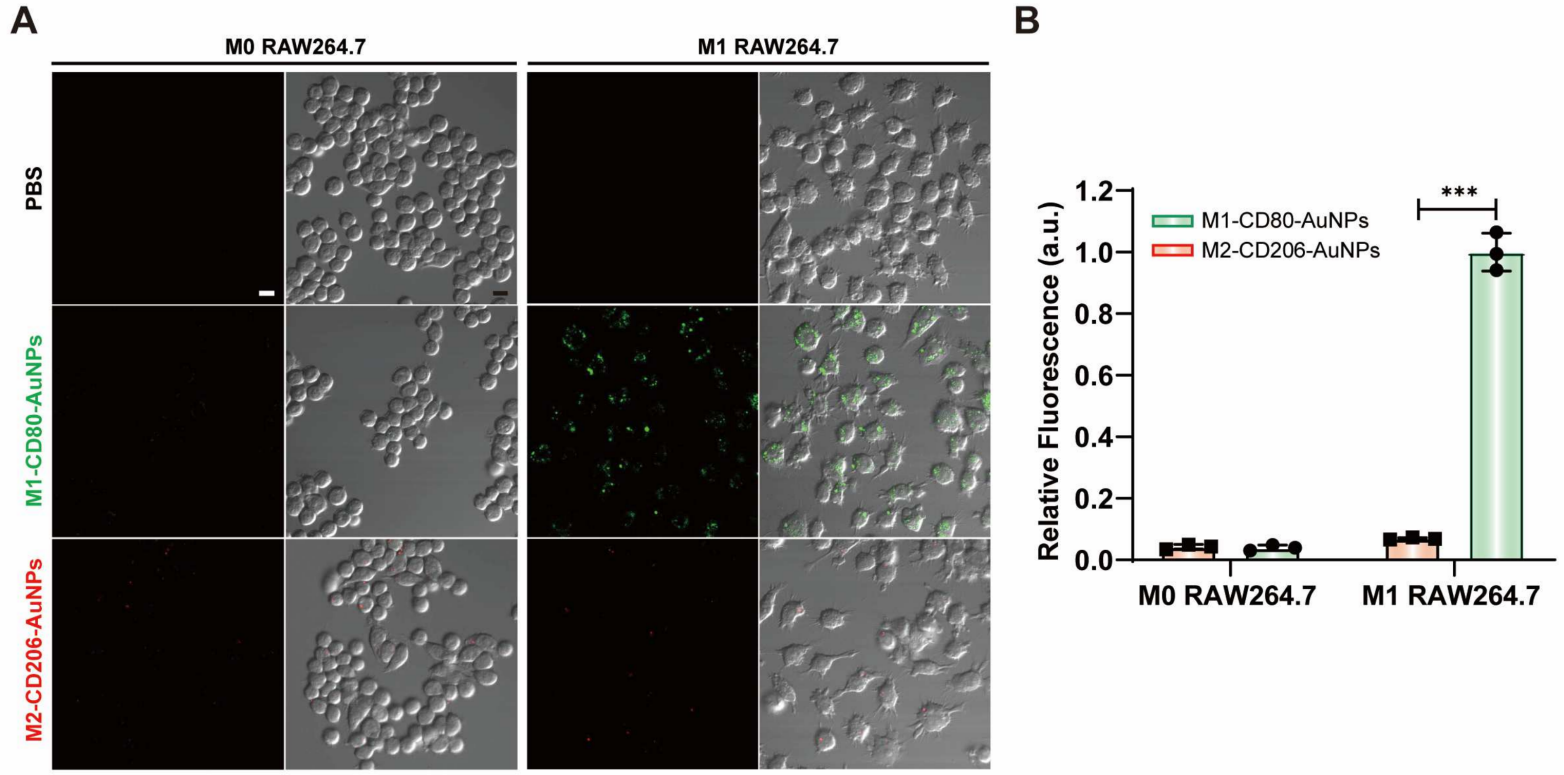
(**A**) Fluorescence images of M0 and M1 RAW264.7 treated with Cy5-labeled fluorescent probes. Scale bar: 10 μm. (**B**) Intracellular fluorescence signals from M0 and M1 RAW264.7 with Cy5-labeled fluorescent mRNA probes. Data are expressed as the mean ± SEM (n = 3) (*** *p* < 0.001).

**Figure 10:**
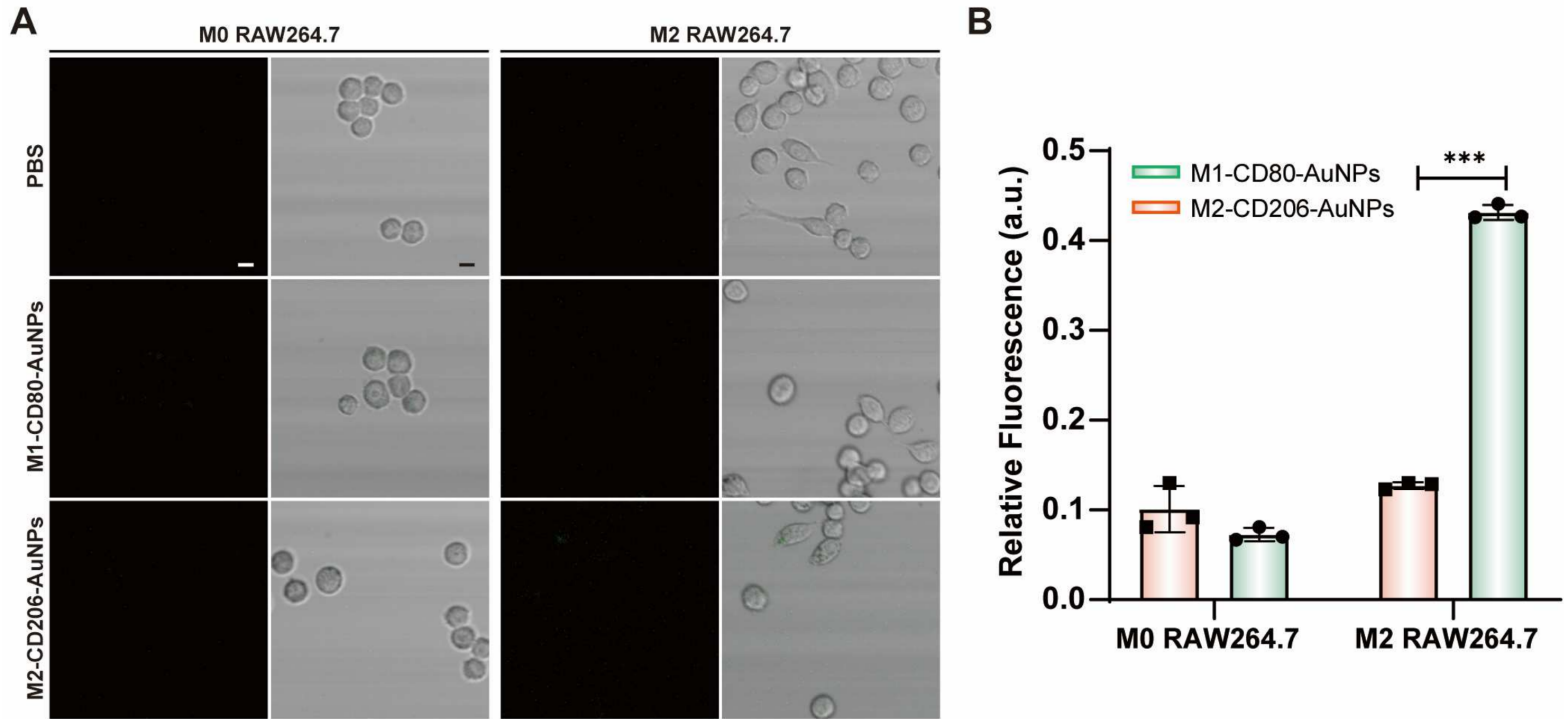
(**A**) Fluorescence images of M0 and M2 RAW264.7 treated with Cy5-labeled fluorescent probes. Scale bar: 10 μm. (**B**) Intracellular fluorescence signals from M0 and M2 RAW264.7 with Cy5-labeled fluorescent mRNA probes. Data are expressed as the mean ± SEM (n = 3) (*** *p* < 0.001).

To evaluate the final live-cell detection effect of polyA20-mediated polarized macrophage fluorescent gold nanoprobes, we selected M1-CD80 and M2-CD206 fluorescent gold nanoprobes for subsequent intracellular imaging experiments. Pure untreated RAW264.7 macrophages were used as a control (labeled as M0), while RAW264.7 macrophages were stimulated and polarized to the M1 phenotype using LPS and M2 phenotype using IL-4, which were used to mimic inflammatory macrophages and anti-inflammatory macrophages respectively in vivo. Equal volumes of PBS with 1 nM final concentration of M1-CD80-AuNPs and M2-CD206-AuNPs fluorescent gold nanoprobes were added to the M0, M1, and M2 macrophage culture systems, respectively. After co-incubation for 3 h, Cy5 fluorescence signals were stimulated by a confocal laser scanning microscope (CLSM) 646 nm laser and the fluorescence confocal images of cells were obtained (Figure 9A and Figure 10A) with quantitative results (Figure 9B and Figure 10B). Under similar conditions, the M1-CD80 fluorescent nanoprobe exhibited strong fluorescent signals only in the irregularly rounded, multitentacled M1 macrophage cell set obtained by LPS stimulation. The M2-CD206 fluorescent nanoprobe only showed strong fluorescence signals in the M2 macrophage group with slender morphology obtained through IL-4 stimulation. Moreover, in the round, aggregate-like M0 macrophages, the M1-CD80-AuNPs and M2-CD206-AuNPs fluorescent nanoprobes in the system produced almost no fluorescent signals.

**Figure 11:**
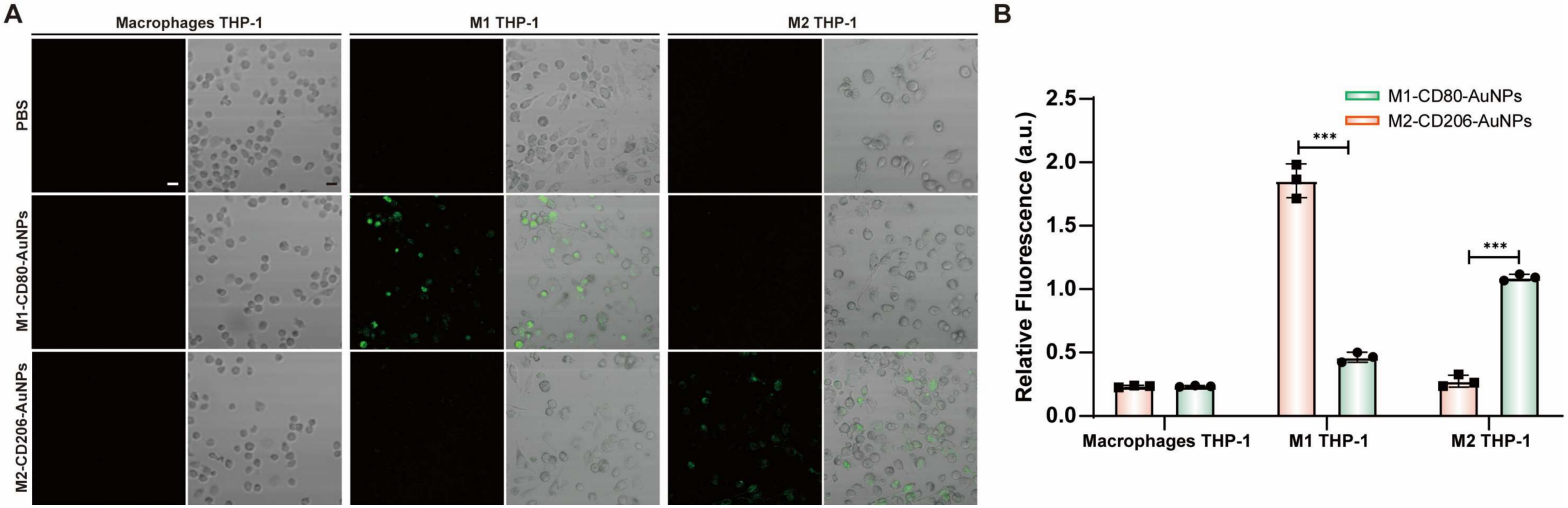
(**A**) Fluorescence images of different phenotypes of macrophages induced by THP-1 treated with Cy5-labeled fluorescent probes. Scale bar: 10 μm. (**B**) Intracellular fluorescence signals from different phenotypes of macrophages induced by THP-1 with Cy5-labeled fluorescent mRNA probes. Data are expressed as the mean ± SEM (n = 3) (*** *p* < 0.001).

To further validate the live-cell detection effect of the fluorescent gold nanoprobes, human-derived cell THP-1 was also applied for intracellular imaging experiments. PMA-treated THP-1 cells were further polarized to the M1 phenotype using LPS and IFN-γ and M2 phenotype using IL-4 and IL-13, while only PMA-treated THP-1 cells were used as control. Fluorescence confocal images (Figure 11A) and quantitative results (Figure 11B) were obtained with a similar experimental method as RAW264.7 macrophages. According to the experimental results, M1-CD80 fluorescent nanoprobes only exhibit strong fluorescence signals in M1 THP-1, and M2-CD206 fluorescent nanoprobes exhibit fluorescence signals in M2 THP-1.

Therefore, polyA20-mediated polarization of macrophage fluorescent gold nanoprobes possesses an excellent ability to endocytose into living cells. This result suggests that the probe can bind specifically to target mRNAs with reporter strand fluorescence recovery, producing optimal fluorescence imaging of living cells. This approach is anticipated to identify inflammatory M1 macrophages and anti-inflammatory M2 macrophages, which can facilitate the diagnosis of macrophage-related diseases in vivo.

## 4. Conclusions

This study developed an efficient fluorescent probe to rapidly identify macrophage polarization phenotypes. The fluorescent probe was built by ligating DNA recognition sequences for macrophage polarization protein markers with polyA20 tails on AuNPs, followed by hybridization with fluorescent reporter sequences modified with Cy5 fluorescent motifs. Using the energy of base complementary pairing, the fluorescent reporter chain could be attached to the CD80 and CD206 mRNA sequences in the macrophage, allowing real-time production of vivid fluorescent signals referring to the status of the macrophage. This design facilitates in situ fluorescence imaging of mRNA in polarized macrophages, offering a robust method to elucidate macrophage complexities. The strategy effectively distinguishes between macrophage phenotypes, providing the medical field with a valuable tool for tracking and monitoring macrophage behavior.

## Figures and Tables

**Table 1:** Sequence information for oligonucleotides used in this study.

Name	Sequence
M1-CD80-A20	TAGCCCTGGCTGTCCTGGAATTTT TAAAAAAAAAAAAAAAAAAAA
M1-CD80-Flare	Cy5-TTCCAGGACAGCCAGGGCTATACAA
M1-CD80-T	TTGTATAGCCCTGGCTGTCCTGGAA
M1-CD80-T-Eror	TTGTATAGCCCTAGCTGTCCTGGAA
M2-CD206-A20	ATTTTGGCTTTTCAACACCCTTTT TAAAAAAAAAAAAAAAAAAAA
M2-CD206-Flare	Cy5-GGGTGTTGAAAAGCCAAAATAGTCT
M2-CD206-T	AGACTATTTTGGCTTTTCAACACCC
M2-CD206-T-Eror	AGACTATTTTGGATTTTCAACACCC
Aptamer	CACCCCACCTCGCTCCCGTGACACTAATGCTA

## Data Availability

The raw and processed data required to reproduce these findings are available from the corresponding author upon reasonable request.
